# Real-World Effectiveness of Budesonide Monotherapy via Pressurized Metered-Dose Inhaler in Steroid-Naive Pediatric Asthma

**DOI:** 10.7759/cureus.108632

**Published:** 2026-05-11

**Authors:** Shabnam, Akanksha D Srivastava, Sarika Gupta, Ajay Verma

**Affiliations:** 1 Pediatrics, Base Hospital, Srinagar, IND; 2 Pediatrics, Career Institute of Medical Sciences, Lucknow, IND; 3 Pediatrics, King George’s Medical University, Lucknow, IND; 4 Respiratory Medicine, King George's Medical University, Lucknow, IND; 5 Pulmonary Critical Care Medicine, King George’s Medical University, Lucknow, IND

**Keywords:** budesonide, childhood asthma, mdi (metered-dose inhaler), pft: pulmonary function test, steroid

## Abstract

Background: Inhaled corticosteroids (ICS) remain the cornerstone of asthma management in children. However, real-world evidence evaluating the effectiveness of budesonide monotherapy in steroid-naive pediatric patients is limited. This study aimed to assess the impact of inhaled budesonide delivered via a pressurized metered-dose inhaler (pMDI) on pulmonary function and asthma control in steroid-naive children with bronchial asthma.

Methods: This prospective observational study was conducted in the Asthma Clinic, Department of Pediatrics, King George’s Medical University, Lucknow, from April 2022 to March 2023 after ethical approval. A total of 108 children aged 5-12 years with physician-diagnosed asthma were screened. After applying inclusion and exclusion criteria, 50 steroid-naive children were enrolled. Steroid-naive status was defined as no prior regular ICS use and no systemic corticosteroid exposure within the preceding three months. All participants received inhaled budesonide monotherapy at a low- to medium-dose of 200-400 μg/day, as per Global Initiative for Asthma (GINA) recommendations, via pMDI with spacer. Baseline and three-month assessments included spirometry (forced expiratory volume in one second (FEV₁), FEV₁% predicted, forced expiratory flow between 25% and 75% (FEF25-75% predicted)), Childhood Asthma Control Test (CACT), Mini Pediatric Asthma Quality of Life Questionnaire (MiniPAQLQ), and Pediatric Asthma Caregiver’s Quality of Life Questionnaire (PACQLQ). Paired comparisons were performed, and p<0.05 was considered statistically significant.

Results: Significant improvement was observed in pulmonary function parameters after three months of budesonide therapy. Post-bronchodilator FEV₁ increased significantly (p<0.001), and FEV₁% predicted improved by 28.7% (p<0.001). FEF25-75% predicted showed a 17.1% increase, indicating improvement in small airway function. Asthma control improved substantially, with 56% of children achieving controlled asthma status at three months compared to none at baseline (p<0.001). Mean CACT scores increased significantly, accompanied by clinically meaningful improvements in MiniPAQLQ and PACQLQ scores (p<0.001 for all comparisons). Budesonide was well tolerated, with no serious adverse effects reported.

Conclusion: Inhaled budesonide monotherapy administered via pMDI significantly improves pulmonary function, asthma control, and quality of life in steroid-naive pediatric asthma patients within three months. These findings suggest that budesonide alone is an effective and reliable controller therapy in real-world clinical settings.

## Introduction

Bronchial asthma is the most prevalent chronic respiratory disorder of childhood, with an estimated global prevalence of 7-10% in children and a rising burden across low- and middle-income countries (LMICs) [1]. It is characterized by chronic airway inflammation, reversible airflow obstruction, and episodic exacerbations, often leading to impaired lung growth, poor school performance, and frequent emergency visits if inadequately controlled [2]. Inhaled corticosteroids (ICS) remain the cornerstone of asthma management in children, as they reduce airway inflammation, improve pulmonary function, and decrease exacerbations [3]. Among ICS, budesonide has been extensively studied and is considered safe and effective even in long-term use, with minimal systemic side effects compared to oral corticosteroids [4].

The delivery device is equally important in ensuring therapeutic efficacy. Pressurized metered-dose inhalers (pMDIs), especially when used with spacers, provide consistent lung deposition, are cost-effective, and are suitable for younger children with poor coordination [5]. Recurrent inflammation leads to airway remodeling, which can impair lung development in childhood and predispose to irreversible obstruction in adulthood if not adequately controlled [6]. Despite this strong evidence, ICS use in India remains suboptimal due to poor adherence, lack of awareness among caregivers, and delayed initiation of controller therapy. Budesonide, in particular, is preferred due to its favorable pharmacokinetics: rapid absorption in the lung, high first-pass metabolism reducing systemic bioavailability, and a demonstrated long-term safety profile [7,8].

Multiple randomized controlled trials have demonstrated that ICS therapy reduces symptoms, improves lung function, and enhances quality of life in asthmatic children. A landmark trial by the Childhood Asthma Management Program (CAMP) showed that continuous budesonide therapy significantly reduced exacerbations and improved control compared to placebo, although it did not alter long-term lung growth [9]. Importantly, early introduction of ICS has been shown to prevent asthma-related school absenteeism and improve exercise tolerance [10]. Budesonide is a potent non-halogenated glucocorticoid that enhances anti-inflammatory gene expression [11].

While randomized controlled trials have established the efficacy of ICS, there is limited real-world evidence evaluating their effectiveness in routine clinical settings, especially among steroid-naive pediatric populations. In the context of this study, real-world effectiveness refers to treatment outcomes observed in routine clinical practice without strict protocol-driven interventions, reflecting typical patient behavior, adherence patterns, and environmental influences.

The present study was designed to assess the real-world effectiveness of inhaled budesonide administered via pMDI in steroid-naive children with bronchial asthma attending a tertiary care center. The primary outcome was improvement in pulmonary function, assessed by forced expiratory volume in one second (FEV₁). Secondary outcomes included improvement in asthma control measured by the Childhood Asthma Control Test (CACT) and quality of life assessed using the Mini Pediatric Asthma Quality of Life Questionnaire (MiniPAQLQ) and Pediatric Asthma Caregiver’s Quality of Life Questionnaire (PACQLQ).

## Materials and methods

Study setting

This prospective observational study was conducted at the Asthma Clinic of the Department of Pediatrics, King George’s Medical University (KGMU), Lucknow, India, over a period of one year from April 2022 to March 2023. Children aged 5-12 years with clinically suspected asthma confirmed by spirometry demonstrating reversible airway obstruction were screened for eligibility as per Global Initiative for Asthma (GINA) guidelines.

Ethical Approval

The study was approved by the Institutional Ethics Committee of KGMU (Approval number 1604, Ref. code: 97th ECMM 2 B-Thesis/P126). Written informed consent was obtained from parents or legal guardians prior to enrollment.

Sample Size Estimation

The sample size was calculated using the formula n=Z²×p×(1-p)/d², where n represents the required sample size, Z is the standard normal variate corresponding to a 95% confidence interval (1.96), p is the estimated prevalence of pediatric asthma based on previous epidemiological studies, and d represents the allowable margin of error [3]. Based on these parameters, a minimum sample size of approximately 50 participants was considered adequate for the present observational study.

Study Population

Children aged 5-12 years presenting with clinically suspected asthma were screened for eligibility. A total of 108 children were screened, of whom 70 met the inclusion criteria. After excluding children with prior corticosteroid exposure, 53 steroid-naive children were enrolled. Inclusion criteria comprised children aged between 5 and 12 years who were diagnosed with persistent bronchial asthma according to GINA guidelines, had not previously received ICS, and were able to perform acceptable spirometry. Children were excluded if they had a history of prior treatment with ICS, acute severe asthma requiring hospitalization, the presence of other chronic respiratory diseases, congenital heart disease, or an inability to perform reliable spirometry.

Diagnosis and severity classification

Diagnosis of asthma was based on clinical history, physical examination, and bronchodilator responsiveness in accordance with GINA guidelines. Spirometry demonstrating reversible airway obstruction was used to support the diagnosis in children able to perform acceptable and reproducible maneuvers. Asthma severity was classified as mild-to-moderate persistent asthma based on GINA criteria.

**Figure 1:**
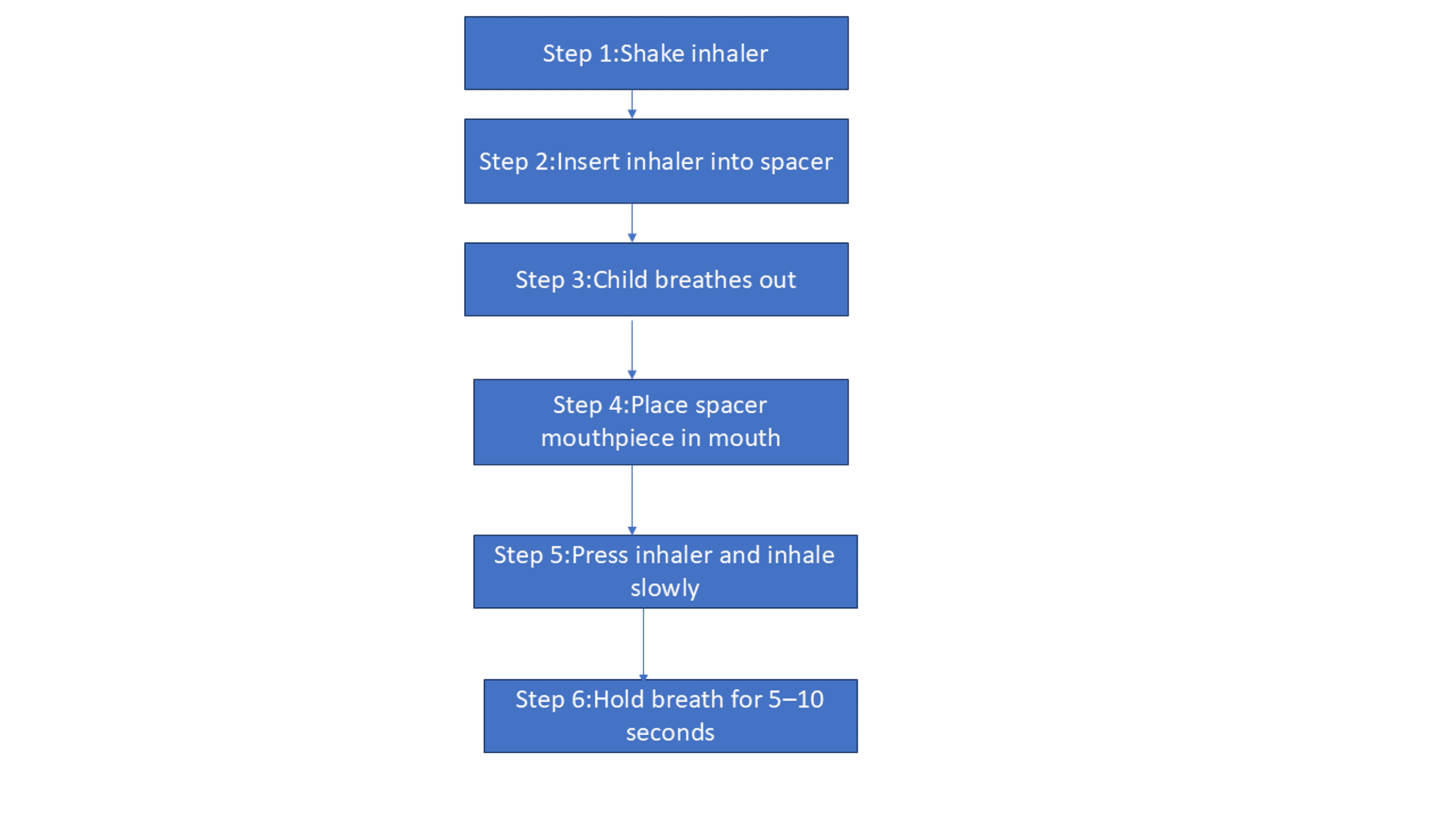
Schematic illustration demonstrating the correct technique for using a pMDI with a spacer in children pMDI, pressurized metered-dose inhaler

All enrolled participants received inhaled budesonide administered via pMDI with a spacer (Figure 1). Budesonide was prescribed at low-to-medium doses ranging from 200 to 400 μg/day, in accordance with GINA Step II-III treatment recommendations, depending on symptom severity and physician assessment. Caregivers and patients were instructed regarding the correct inhaler technique using a spacer device, and adherence to therapy was reinforced during follow-up visits.

Pulmonary function was assessed using spirometry, measuring parameters including FEV₁ and forced vital capacity (FVC), expressed as percentages of predicted values. Spirometry was performed using standardized equipment with calibration and reproducibility ensured according to American Thoracic Society/European Respiratory Society (ATS/ERS) guidelines. Multiple attempts and appropriate coaching were provided to obtain acceptable maneuvers. In younger children, spirometry findings were interpreted along with clinical assessment. Asthma control was evaluated using the CACT [12], a validated questionnaire used to assess symptom control in children with asthma. Scores ranged from 0 to 27, with higher scores indicating better asthma control. Health-related quality of life was assessed using a validated pediatric asthma quality-of-life questionnaire described in the literature [13,14], evaluating domains such as symptoms, activity limitation, and emotional function. Participants were followed for three months after initiation of therapy. Repeat spirometry and clinical assessment were performed at the end of the follow-up period. The primary outcome was a change in pulmonary function parameters. Secondary outcomes included change in asthma control and quality-of-life scores.

Statistical analysis was performed using IBM SPSS Statistics for Windows, Version 26.0 (IBM Corp., Armonk, NY, USA; released 2019). Continuous variables were expressed as mean±standard deviation, while categorical variables were presented as frequency and percentage. Prior to applying parametric tests, the assumption of normality of continuous variables was assessed using the Shapiro-Wilk test. As the data were found to be approximately normally distributed, paired t-tests were used to compare baseline and follow-up values. For variables not meeting normality assumptions, appropriate nonparametric tests would have been considered. A p-value<0.05 was considered statistically significant.

## Results

**Figure 2:**
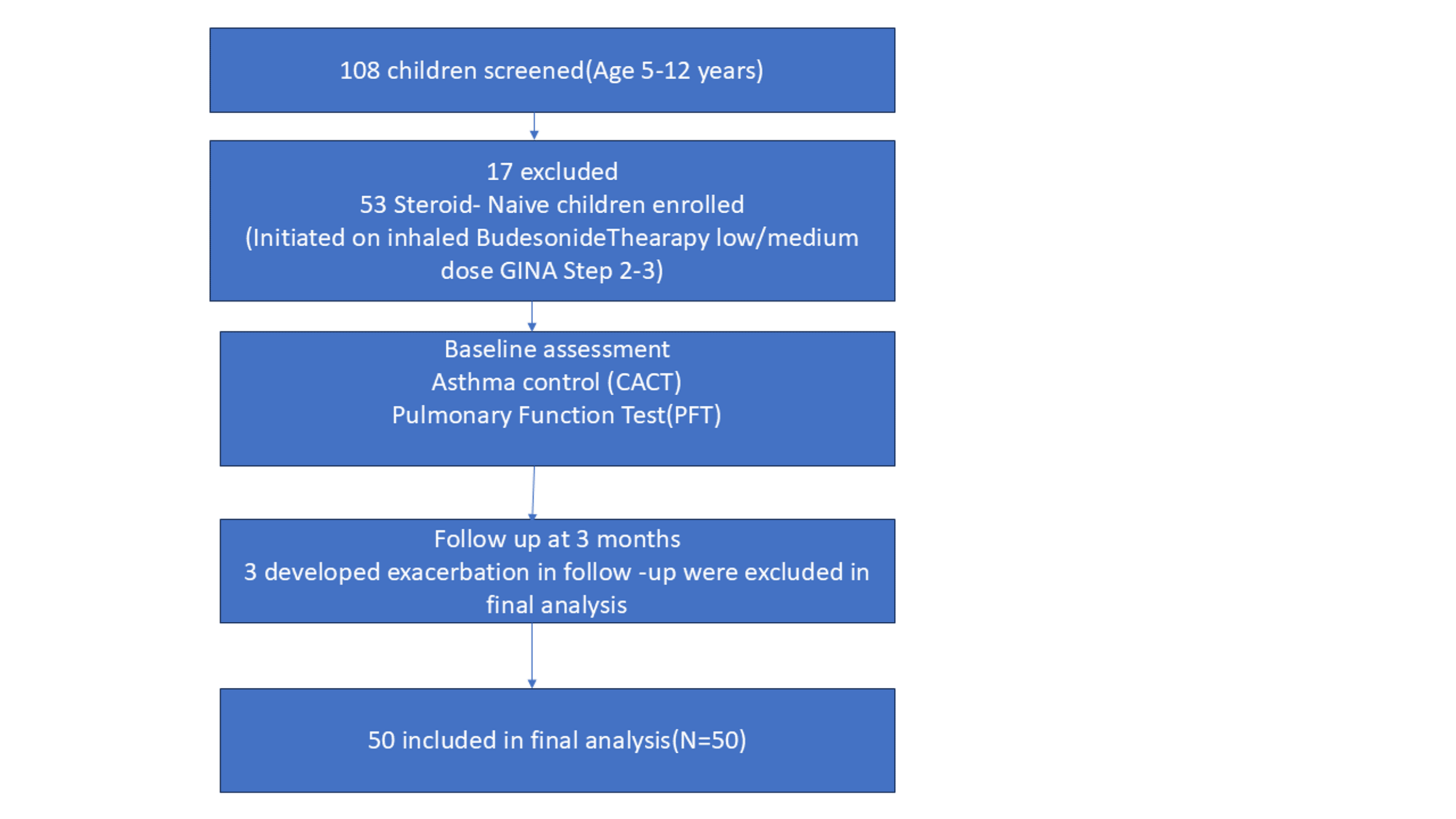
Flow diagram showing screening, enrollment, follow-up, and final analysis of steroid-naive children receiving inhaled budesonide therapy GINA, Global Initiative for Asthma; CACT, Childhood Asthma Control Test

A total of 108 children aged 5-12 years were screened during the study period. Seventy met the initial inclusion criteria, of whom 17 were excluded due to recent systemic or ICS use within the preceding three months. Fifty-three steroid-naive children were initiated on inhaled budesonide therapy ranging from 200 to 400 μg/day according to GINA Step 2-3 recommendations. During follow-up, three children developed exacerbations and were excluded from pulmonary function analysis, as exacerbations could significantly influence outcome measures and interfere with the assessment of maintenance therapy effectiveness. Fifty children completed the three-month follow-up and were included in the final analysis (Figure 2).

The mean age of the participants was 10.9±3.4 years, and 31 (62%) were male. The mean weight and height were 29.32±11.35 kg and 130.18±14.87 cm, respectively. Twenty-seven (54%) children resided in urban areas. Environmental risk factors included residence within 1 km of traffic in 15 (30%) children and nearby industrial exposure in 11 (22%). Exposure to second-hand smoke was reported in 17 (34%) participants. Allergic rhinitis was present in 27 (54%) children, atopic dermatitis in 7 (14%), and allergic conjunctivitis in 10 (20%). A family history of atopy was reported in 17 (34%) participants. Dampness in the house was observed in 11 (22%) children, and cockroach exposure in 7 (14%) (Table 1).

**Table 1:** Basic demographic and clinical characteristics of steroid-naive children with uncontrolled asthma (N=50) Values are presented as mean±SD for continuous variables and number (percentage) for categorical variables. GERD, gastroesophageal reflux disease; LPG, liquefied petroleum gas

Characteristics	N=50
Age (years), mean±SD	10.9±3.4
Male, n (%)	31 (62.0)
Weight (kg), mean±SD	29.32±11.35
Height (cm), mean±SD	130.18±14.87
Urban residence, n (%)	27 (54.0)
Distance of residence from traffic ≤1 km, n (%)	15 (30.0)
Industrial factory in nearby area, n (%)	11 (22.0)
Presence of separate cooking space, n (%)	43 (86.0)
Exclusive use of LPG for cooking, n (%)	46 (92.0)
Exposure to second-hand cigarette smoke, n (%)	17 (34.0)
Allergic rhinitis, n (%)	27 (54.0)
Atopic dermatitis, n (%)	7 (14.0)
Allergic conjunctivitis, n (%)	10 (20.0)
GERD, n (%)	3 (6.0)
Family history of atopy, n (%)	17 (34.0)
Animals (dog/cat) in house, n (%)	10 (20.0)
Dampness in house, n (%)	11 (22.0)
Cockroach in house, n (%)	7 (14.0)

**Figure 3:**
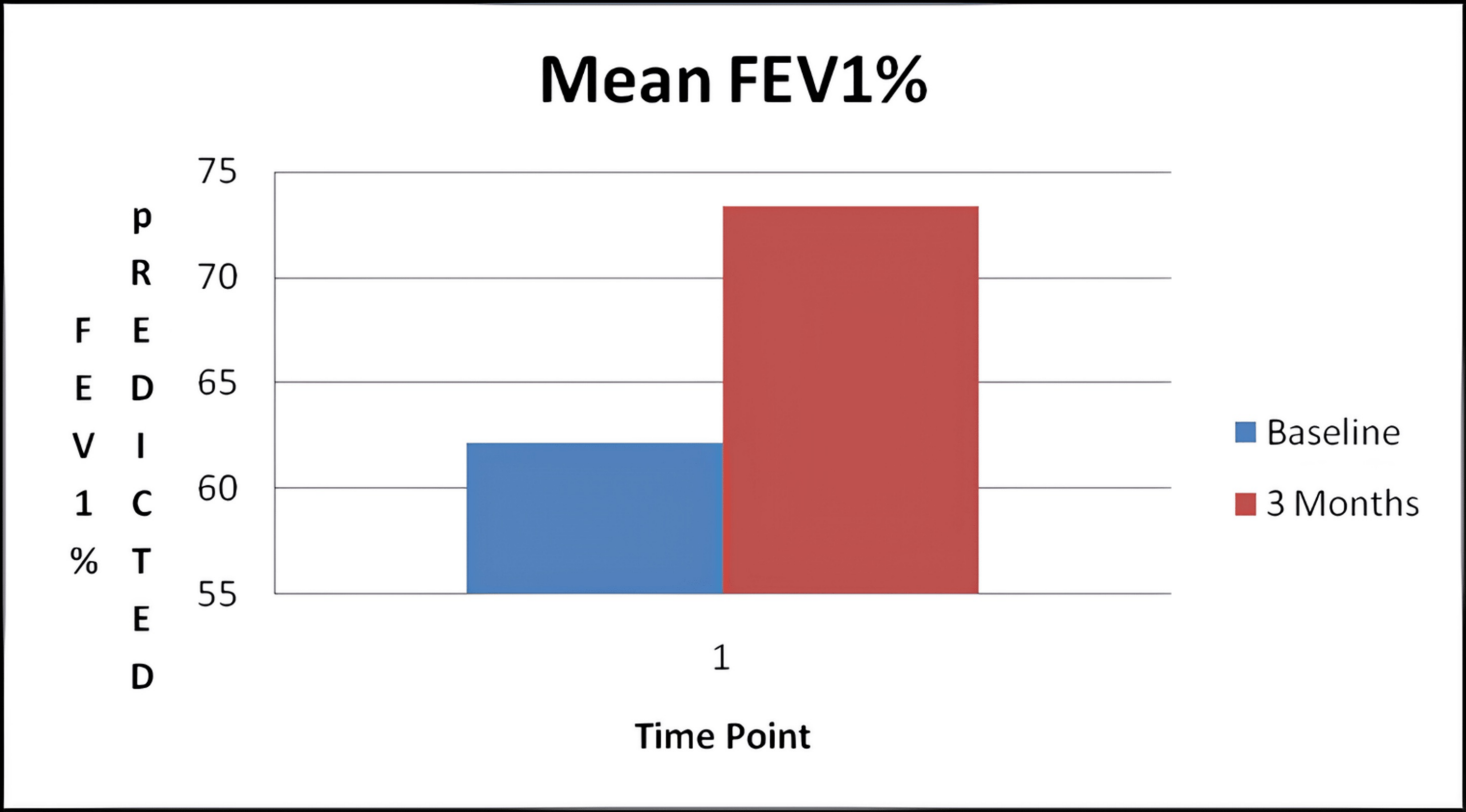
Bar chart comparing mean FEV₁ (% predicted) at baseline and three months after inhaled budesonide therapy FEV₁, forced expiratory volume in one second

After three months of inhaled budesonide therapy, significant improvement was observed in pulmonary function parameters. The mean FEV₁/FVC ratio increased from 65.2±5.6 at baseline to 74.1±5.2 at follow-up (p<0.001). The mean pre-bronchodilator FEV₁ increased from 1.42±0.25 L to 1.58±0.23 L (p<0.001), while post-bronchodilator FEV₁ improved from 1.55±0.27 L to 1.72±0.25 L (p<0.001). Similarly, FEV₁% predicted improved from 62.1±4.9 to 73.4±5.7 (p<0.001). The FEF25-75% predicted increased from 59.8±9.4 to 72.6±10.2 (p=0.001). These findings indicate significant improvement in airway function following three months of inhaled budesonide therapy (Table 2, Figure 3).

**Table 2:** Mean change in pulmonary function test parameters after three months of inhaled budesonide controller therapy (N=50) Values are expressed as mean±SD. Comparison between baseline and follow-up measurements was performed using the paired t-test. A p-value<0.05 was considered statistically significant. FEV₁, forced expiratory volume in one second; FVC, forced vital capacity; FEF25-75%, forced expiratory flow between 25% and 75% of forced vital capacity.

Pulmonary function test parameters	Baseline (mean±SD)	Follow up (mean± SD)	Mean difference (% change)	p-value
FEV₁/FVC ratio	65.2±5.6	74.1±5.2	8.9 (13.6%)	<0.001
Pre-bronchodilator FEV₁ (L)	1.42±0.25	1.58±0.23	0.16 (11.3%)	<0.001
Post-bronchodilator FEV₁ (L)	1.55±0.27	1.72±0.25	0.17 (11%)	<0.001
FEV₁​​​​​​​% predicted	62.1±4.9	73.4±5.7	11.3 (18.2%)	<0.001
FEF25-75% predicted	59.8±9.4	72.6±10.2	12.8 (21.4%)	0.001

**Figure 4:**
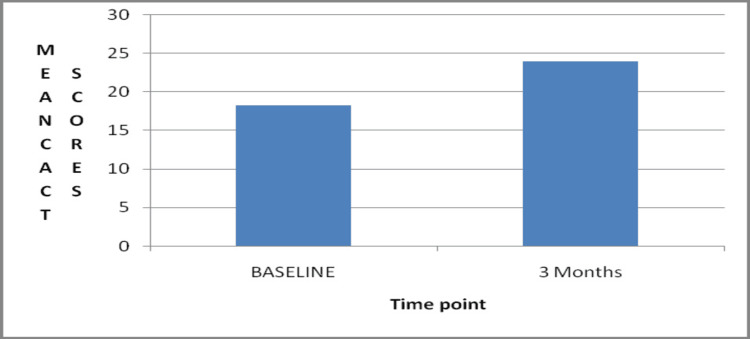
Bar chart comparing CACT scores at baseline and after three months of inhaled budesonide therapy CACT, Childhood Asthma Control Test

Significant improvement was also observed in asthma control and quality-of-life scores. The mean CACT score increased from 18.3±2.5 at baseline to 24.0±2.2 at follow-up (p<0.001). The MiniPAQLQ score improved from 4.2±0.8 to 5.6±0.7 (p<0.001), while the PACQLQ score increased from 4.3±0.9 to 5.8±0.8 (p<0.001) after three months of therapy (Table 3, Figure 4).

**Table 3:** Mean change in CACT score, MiniPAQLQ score, and PACQLQ score after three months of inhaled budesonide controller therapy (N=50) Values are expressed as mean±SD. Comparisons between baseline and follow-up scores were performed using the paired t-test. CACT: Childhood Asthma Control Test; MiniPAQLQ: Mini Pediatric Asthma Quality of Life Questionnaire; PACQLQ: Pediatric Asthma Caregiver Quality of Life Questionnaire

Scores	Baseline (mean±SD)	Follow up (mean±SD)	Mean difference (% change)	p-value
CACT	18.3±2.5	24.0±2.2	+5.7 (+31.1%)	<0.001
MiniPAQLQ	4.2±0.8	5.6±0.7	+1.4 (+33.3%)	<0.001
PACQLQ	4.3±0.9	5.8±0.8	+1.5 (+34.9%)	<0.001

## Discussion

The present study demonstrates that budesonide administered as monotherapy via pMDI produces significant clinical and functional improvement in steroid-naive children with bronchial asthma. Within three months of initiation, substantial gains were observed in post-bronchodilator FEV₁, FEV₁% predicted, FEV₁/FVC ratio, and small airway indices, including FEF25-75%. In addition, asthma control scores improved markedly, and exacerbation frequency declined significantly. These findings reinforce the central role of ICS monotherapy as the foundation of pediatric asthma management.

Our findings are consistent with large international trials demonstrating the efficacy of budesonide in improving lung function and reducing symptoms in pediatric asthma. While combination therapies such as budesonide-formoterol and maintenance and reliever therapy (MART) regimens have been shown to provide rapid bronchodilation and reduce severe exacerbations in selected populations, it is important to recognize that anti-inflammatory control remains the cornerstone of asthma management. In steroid-naive children with mild-to-moderate persistent asthma, initiation of adequate-dose ICS alone may be sufficient to achieve meaningful clinical control without the immediate need for long-acting bronchodilator combinations.

Pedersen et al. reported significant improvements in symptom control and pulmonary function in children treated with budesonide compared with placebo, highlighting its role as an effective anti-inflammatory controller therapy [15]. Similarly, Baker et al. [16] demonstrated that budesonide inhalation suspension administered once or twice daily resulted in significant improvement in asthma symptoms and lung function in young children with persistent asthma, reinforcing the effectiveness of ICS monotherapy in pediatric populations.

The landmark CAMP trial by the CAMP Research Group further established that long-term budesonide therapy significantly improved asthma control, airway responsiveness, and reduced exacerbations compared to non-anti-inflammatory therapy [9]. Although combination therapies were not the primary focus of CAMP, the study clearly supported ICS as the foundational controller therapy in children.

In a systematic review and meta-analysis, Castro-Rodriguez and Rodrigo concluded that ICS significantly reduced exacerbations and improved symptoms in infants and preschool children with recurrent wheezing and asthma compared with placebo or no ICS therapy [17]. These findings align closely with our study, where steroid-naive children demonstrated marked improvement in both large airway indices (post-FEV₁, FEV₁% predicted, FEV₁/FVC ratio) and small airway parameters (FEF25-75%), along with significant improvement in asthma control scores.

Furthermore, comparative analyses have suggested that while the addition of long-acting β₂-agonists may offer faster bronchodilation in selected patients, optimized ICS monotherapy remains highly effective in achieving disease control in mild-to-moderate pediatric asthma. Pearlman et al. showed that although budesonide/formoterol provided additional bronchodilatory benefit, budesonide alone still produced substantial improvement in lung function and symptom control [18].

Taken together, these studies support the findings of the present research, which demonstrate that appropriately dosed budesonide monotherapy via pMDI can produce clinically meaningful improvement in lung function, asthma control, and exacerbation reduction in steroid-naive children. In resource-limited settings, where cost and inhaler complexity are important considerations, budesonide alone remains a highly effective and practical first-line controller therapy.

Strengths of the study

This study prospectively evaluated steroid-naive children with moderate persistent asthma, allowing assessment of the true therapeutic impact of budesonide without confounding from prior corticosteroid use. Both objective spirometric parameters and validated asthma control and quality-of-life tools were used, providing a comprehensive evaluation of treatment response. Conducted in a real-world tertiary care setting with standardized spirometry techniques, the findings enhance the clinical relevance and generalizability of budesonide monotherapy as an effective first-line controller therapy.

Limitations

This study has several limitations that should be considered while interpreting the findings. First, the absence of a control or comparison group limits the ability to establish causal relationships between inhaled budesonide therapy and observed improvements; the results should therefore be interpreted as associations within a real-world clinical context. Second, the relatively small sample size and single-center design may limit the generalizability of the findings to broader populations.

Third, the short follow-up duration of three months restricts the assessment of long-term outcomes, including sustained asthma control, exacerbation frequency, and disease progression. Additionally, the improvements observed may partly reflect regression to the mean, natural variability of asthma symptoms, or enhanced adherence and inhaler technique over time (Hawthorne effect).

Selection bias is also possible, as only children capable of performing acceptable spirometry were included, potentially excluding younger or less cooperative patients. Furthermore, although potential confounding factors such as environmental exposures, adherence, and comorbidities were recorded, statistical adjustment for these variables was limited.

Finally, adherence assessment was based primarily on caregiver reporting rather than objective measures, which may introduce reporting bias.

Additionally, the study evaluated budesonide monotherapy without including a comparison group receiving MART or other combination ICS-formoterol regimens recommended in recent asthma management strategies, such as those proposed by GINA. The absence of a comparative treatment arm limits the ability to directly assess the relative effectiveness of different controller strategies.

Future multicenter randomized controlled studies with larger sample sizes and longer follow-up periods are needed to compare budesonide monotherapy with MART-based regimens and other guideline-recommended treatment approaches in pediatric asthma.

Future prospects

In this prospective observational study conducted in a real-world clinical setting, inhaled budesonide monotherapy administered via a pMDI was associated with significant improvements in pulmonary function, asthma control, and quality of life in steroid-naive children with bronchial asthma over a three-month follow-up period.

However, given the single-arm design and absence of a control group, these findings should be interpreted as associations rather than evidence of causality. The observed improvements may also be influenced by factors such as natural disease variability, improved adherence, and optimization of inhaler technique.

Further large-scale, controlled studies with longer follow-up are warranted to confirm these findings and to better establish the long-term effectiveness and comparative benefits of inhaled budesonide in pediatric asthma management.

Future studies should focus on multicenter trials with larger sample sizes and longer follow-up periods to further validate the effectiveness of ICS therapy in pediatric asthma. Comparative studies evaluating budesonide monotherapy versus combination regimens such as MART may provide additional insights into optimal treatment strategies for steroid-naive children.

In addition, future research may explore the role of environmental factors, adherence to inhaler technique, and caregiver education in improving asthma control and quality of life outcomes in children. Long-term follow-up studies assessing exacerbation rates, hospitalization rates, and sustained pulmonary function improvement would further strengthen the evidence base for guideline-directed asthma management in real-world clinical settings.

## Conclusions

Inhaled budesonide monotherapy administered via pMDI significantly improves pulmonary function, asthma control, and quality of life in steroid-naïve pediatric asthma patients within three months. These findings suggest that budesonide alone is an effective and reliable controller therapy in real-world clinical settings.
